# The Aldersgate-Street Dispensary

**Published:** 1833-10

**Authors:** 


### III. THE ALDERSGATE STREET DISPENSARY.

It has been enacted by this governors of this dispensary, that a governor shall be entitled to vote at an election by paying his subscription a week previously; or, in other words, that the election of a medical officer is to be decided, not by his merits, but his purse. Very charitable people might suppose that this blunder of the governors arose from simple fatuity, and that they did not see the inevitable consequences of this absurd law; but it appears pretty clearly, from some babble of the committee, reported in the newspapers, that they knew its tendency, and were delighted with it. Moreover, the alphabet of common sense was explained to the meeting at some length by the medical officers, who were outvoted, however, by a considerable majority. They have all resigned in consequence, and carry with them the sympathy and good wishes of every man of sense, honour, or feeling. As we are not writing for children, we think all comment on this affair quite superfluous. It is rare indeed to see a dispute in which one party contrives to put itself so completely and indefensibly in the wrong, as the Aldersgate street committee.

The following address appears to us worthy of insertion in our pages:

"Gentlemen: At this interesting period in the state of our profession, and especially of the government of medical charities, we should be guilty of the most culpable apathy and indifference did we not hasten to express the high satisfaction with which we have viewed your conduct on a late occasion; did we not testify how completely our feelings and sentiments are in unison with your own; did we not publicly record our warmest admiration of that brilliant example of integrity and independence which you have exhibited to your medical brethren.

"Whatever sacrifice of feeling or of interest has been made, you have the consolatory approval of upright consciences. You have acted consistently and like honourable men. You have discharged an important duty both to medical practitioners and to society at large. You have nobly withstood a most disgraceful attempt to degrade your profession, and to convert an health-giving charity into an infected source of misery and wretchedness; and, whilst you enjoy the praise of every virtuous and enlightened mind, it may perhaps prove a further gratification to know that your medical brethren in particular are fully alive to the moral force of your example, that they appreciate its worth, and are resolved its memory shall not die.

"Gentlemen: we hail this example, the first practical inroad on a protracted and systematic abuse of medical charities, as the harbinger of a general and efficient reform in those charities; we hail it as the forerunner of an approaching day, when the Legislature shall rescue them from longer prostitution of their legitimate ends,—a day, when the neglected objects of science and the aims of general utility and benevolence shall happily be united, when nor personal interest, nor gold, nor any other corrupt means whatever, shall give notoriety to the officer of an hospital or dispensary, but when talent and knowledge alone, approved by public competition, shall be the test of fitness for office in such institutions.

"The folly which compelled your withdrawal from the charity you upheld foresaw not the consequence about to follow. Its sordid calculations of gain did not reckon that, with the loss of your labours and skill, the very charity you served would cease to exist. For, where shall be found the men reckless enough to succeed you? The very attempt would brand their characters with all that is low, degraded, and debased.

"Accept, gentlemen, this tribute of our gratitude; we deeply feel the obligation under which you have laid the whole profession. Assuredly, the members of that profession will not forget a lesson so disinterested and elevated as that which you have given for their instruction, signalized too as it is, by names distinguished in the scientific and literary history of their country.

"We remain, gentlemen, with the greatest respect and admiration,

"Corden Thompson, M.D., Physician to the Sheffield General Infirmary.
Wilson Overend, Surgeon to the Sheffield General Infirmary.
Henry Paul Harewood, M.D., Physician to the Sheffield Gen. Dispensary.
John Green.
George Calvert Holland, M.D., Physician to the Sheffield Gen. Infirm.
Henry Thomas, Surgeon to the Sheffield Public Dispensary.

| | | |
|---|---|---|
| George Turton, | R. G. Holland, | Thomas Reade, |
| Henry Hardy, | Charles Eadon, | Joseph Law, |
| Joseph Ingall, | R. S. Taylor, | John Carr, |
| James Wild, | G. Reedal, | George Wm. Clark, |
| James Ray, | James F. Wright, | Edward Thompson, |
| Henry Boultbee, F.L.S. | Edward Gillott, | W. Lennard, M.D. |
| Knowlton Wilson, | John Hall, | Samuel Gaegory, |
| John Pearce Lewis, | John Foster, | John Turton, |

Joseph Riley,
William Staniforth, Senior Surgeon to the Infirmary,
Wright Wilson,
Henry Jackson, Junior Surgeon to the Sheffield General Infirmary,
James Walker,
R. Ernest, M.D., House Surgeon to the Sheffield General Infirmary.

| | | |
|---|---|---|
| John Pearson Shaw, | W. Favell, | John Haxworth, Surgeon, |
| William Jackson, | Francis Pearson. | |

*Sheffield; September* 22."

In our next number we shall discuss the subject of Dispensaries, including not only those maintained by subscriptions, but the self-supporting ones.*

* Even those who have never been initiated into editorial mysteries must know, by the apologies ever attending the appearance of a new periodical, how difficult, (we might almost say, how impossible,) it is for an editor to realize even his own expectations in his first number. We trust that the good-natured reader will excuse especially the meagreness of our Medical Politics. The ordinary author rides in his private carriage, and may set off when he pleases: the editor drives a stagecoach, and must be ready at the appointed moment, whether the vehicle be full or empty.

